# Trends in chlamydia and gonorrhea positivity among heterosexual men and men who have sex with men attending a large urban sexual health service in Australia, 2002-2009

**DOI:** 10.1186/1471-2334-11-158

**Published:** 2011-06-05

**Authors:** Lenka A Vodstrcil, Christopher K Fairley, Glenda Fehler, David Leslie, Jennifer Walker, Catriona S Bradshaw, Jane S Hocking

**Affiliations:** 1 Melbourne School of Population Health, The University of Melbourne, Vic, Australia; 2 Melbourne Sexual Health Centre, Alfred Hospital, Vic, Australia; 3 Victorian Infectious Diseases Reference Laboratory, Vic, Australia; 4 Department of Epidemiology and Preventive Medicine, Monash University, Vic, Australia; 5 Centre for Women's Health, Gender and Society, Melbourne School of Population Health, The University of Melbourne, Parkville, Vic, Australia

**Keywords:** Chlamydia, Gonorrhea, Men who have sex with men, Heterosexual men, Positivity

## Abstract

**Background:**

To determine whether chlamydia positivity among heterosexual men (MSW) and chlamydia and gonorrhea positivity among men who have sex with men (MSM), are changing.

**Methods:**

Computerized records for men attending a large sexual health clinic between 2002 and 2009 were analyzed. Chlamydia and gonorrhea positivity were calculated and logistic regression used to assess changes over time.

**Results:**

17769 MSW and 8328 MSM tested for chlamydia and 7133 MSM tested for gonorrhea. In MSW, 7.37% (95% CI: 6.99-7.77) were chlamydia positive; the odds of chlamydia positivity increased by 4% per year (OR = 1.04; 95% CI: 1.01-1.07; p = 0.02) after main risk factors were adjusted for. In MSM, 3.70% (95% CI: 3.30-4.14) were urethral chlamydia positive and 5.36% (95% CI: 4.82-5.96) were anal chlamydia positive; positivity could not be shown to have changed over time. In MSM, 3.05% (95% CI: 2.63-3.53) tested anal gonorrhea positive and 1.83% (95% CI: 1.53-2.18) tested pharyngeal gonorrhea positive. Univariate analysis found the odds of anal gonorrhea positivity had decreased (OR = 0.93; 95% CI: 0.87-1.00; p = 0.05), but adjusting for main risk factors resulted in no change. Urethral gonorrhea cases in MSM as a percentage of all MSM tested for gonorrhea also fell (p < 0.001).

**Conclusions:**

These data suggest that chlamydia prevalence in MSW is rising and chlamydia and gonorrhea prevalence among MSM is stable or declining. High STI testing rates among MSM in Australia may explain differences in STI trends between MSM and MSW.

## Background

*Chlamydia trachomatis *(chlamydia) and *Neisseria gonorrhea *(gonorrhea) diagnosis rates have increased in Australia over the last decade from 88.6 per 100,000 population in 2002 to 286.6 per 100,000 in 2009 for chlamydia and from 30.7 per 100,000 in 2002 to 37.0 per 100,000in 2009 for gonorrhea [1]. While chlamydia diagnoses have increased in both men and women, increases in gonorrhea have been largely confined to men who have sex with men (MSM) [2].

Surveillance data do not provide a valid measure of either the prevalence or change in chlamydia in the general population as over 80% of cases are asymptomatic [3], and testing rates are low in Australia (less than 12% of 15-24-year-old women and less than 5% of similarly aged men) [4]. A recent analysis of a large sexual health clinic in Australia, found that chlamydia positivity increased by about 13% per year in women [5]. A similar analysis of chlamydia positivity among MSW in Australia is yet to be published.

Sexually transmitted infection (STI) notifications among MSM have increased over the last decade in Australia [6] and internationally [7-9]. In response, there has been a push to increase STI testing with new testing guidelines introduced in 2002 [10]. These guidelines recommend that MSM should have annual chlamydia, syphilis, gonorrhea and HIV tests and men at higher risk of STIs (e.g. more than six partners in the last six months) should be tested more frequently [10]. Since the release of the guidelines, self reported STI testing rates among MSM have increased [11]. In a survey of MSM conducted annually in the State of Victoria, Australia, the proportion of HIV negative MSM reporting an annual STI test (excluding blood tests) has risen from 46% in 2003 to 56% in 2009 (p < 0.01) [11]. It is unclear what STI testing coverage is needed to achieve a reduction in STI transmission. National STI surveillance data are unable to answer this question because sexual orientation is not routinely collected. Analyses of sentinel clinic data where sexual orientation is recorded could help to monitor positivity over time.

In this paper we present the results of an analysis of the computerized client records for men attending a large urban sexual health clinic in Australia between 2002 and 2009. This analysis aimed to determine whether chlamydia positivity has changed over time in MSW. We also aimed to determine whether increased STI testing was associated with changes in chlamydia or gonorrhea positivity among MSM.

## Methods

### Study Population and Data Collection

This was a retrospective review of computerized records between 1^st ^of July 2002 and 30^th ^of June 2009 at the Melbourne Sexual Health Centre (MSHC), the principle public STI clinic in Melbourne, the capital city of the State of Victoria, Australia. The clinic conducts over 30,000 consultations annually and provides free HIV and STI testing, using a walk in triage service for new clients attending the centre. Individual computerized records include basic demographic information such as age and gender; behavioral information such as number of male and female sexual partners in the last three and twelve months, and condom use; and clinical information including the presence or absence of genital symptoms, and whether or not the client reports contact with chlamydia and/or gonorrhea. Symptoms can be chlamydia or gonorrhea associated such as discharge or pelvic pain, or unrelated symptoms such as genital ulcers or genital lumps. The computerized record does not specify the type of symptoms.

MSW are defined as men who have had sex with a woman only in the previous 12 months and MSM are defined as men who have had sex with another man in the previous 12 months. Only clients attending MSHC for the first time during the time period and were tested for chlamydia if they were MSW or chlamydia and/or gonorrhea if they were MSM, were included in the analysis.

MSHC policy throughout the time period the study, was to offer first pass urine or urethral swabs for chlamydia to all MSW presenting to the clinic for the first time and to test MSM in accordance with the STI testing guidelines [10]. These guidelines recommend pharyngeal swabs for gonorrhea, first pass urine for chlamydia and anal swabs for both gonorrhea and chlamydia if they have had anal sex. Both policies remained consistent and unchanged throughout the study period.

We also report the number of cases of urethral gonorrhea diagnosed at MSHC during the study period in MSM. Screening for urethral gonorrhea in men without symptoms is not recommended in Australia because asymptomatic urethral gonorrhea is extremely rare [12]. In order to investigate trends in urethral gonorrhea in MSM at MSHC, we calculated the proportion of urethral infections among all MSM attending MSHC who had had a test for gonorrhea at any site during the time period.

In order to examine whether changes observed at MSHC are consistent with changes observed elsewhere in Australia, we analyzed the number of chlamydia and gonorrhea tests undertaken by the Victorian Infectious Diseases Reference Laboratory (VIDRL) over the same time interval. VIDRL is a public health laboratory that provides STI testing services to two large general practice clinics that have a high case load of MSM. The number of anal and pharyngeal swabs tested and number of positive diagnoses was obtained and chlamydia and gonorrhea positivity calculated to explore changes over time. Men who tested more than once within any two-week period were only included once in the analysis and the sexual orientation of the patients were not known. For the purpose of this additional analysis, we assumed that any anal or pharyngeal swabs tested were from MSM. We also obtained the number of gonorrhea diagnoses notified to the Victorian Department of Health during the study period. Gonorrhea diagnoses are required by law to be notified.

This study was a clinical audit that included only de-identified data. According to the national guidelines, an ethics application is not required when there is no risk involved [13].

### Laboratory Methods

At MSHC, all specimens were analyzed for chlamydia using BD ProbeTec Strand Displacement Amplification NAA assay [5]. Anal and pharyngeal swabs for gonorrhea testing were directly plated at the time of specimen collection onto Thayer Martin medium and immediately delivered to the on-site laboratory for culture.

At VIDRL, a number of NAA assays for detection of both chlamydia and gonorrhea were used over the study period. Both dry swabs (for PCR only) and swabs in charcoal Amies transport medium (for culture and/or PCR) were collected from anal and pharyngeal sites. Initially, the Roche Cobas Amplicor^® ^CT/NG PCR Assay was used to test total DNA extracts prepared using A Roche™ MagNaPure or Corbett™ automated DNA extractor. Given the specificity problem of the Amplicor assay for the detection of gonorrhea [14], all initial gonorrhea positive results were confirmed by a second in-house PCR assay, the first to be used targeting 16S rRNA gene, the second the cppB gene, and the third the polA pseudogene. Later, the Amplicor assay was replaced by the Abbott™ m2000 TaqMan-based PCR system which includes an automated total DNA extraction step. For gonorrhea culture, swabs in charcoal Amies transport medium were plated out on New York City Agar and Chocolate or Horse-blood agar (depending on site) and incubated at 37°C in 5% CO_2_. The plates were read daily for up to 4 days and isolates identified by conventional phenotypic methods.

### Statistical Analyses

Chlamydia and gonorrhea positivity (the proportion of men tested who were positive) estimates and 95% confidence intervals were calculated using exact methods. Logistic regression was used to assess associations between chlamydia or gonorrhea positivity and year of test adjusting for demographic, clinical, and sexual behavioral risk factors. Contact with chlamydia or gonorrhea and symptomatic presentations of either infection were collapsed together for pragmatic purposes as demonstrated previously [5]. Number of partners in the last 3 months was strongly correlated with the number of partners in the last 12 months and condom use in the last 3 months was strongly correlated with condom use in the last 12 months. As a result, only one of the two number of partners variables and one of the two condom use variables was included in each regression model; these were selected based on the likelihood ratio test (p < 0.05) and varied between analyses. Chi-squared analysis of trends was used to assess changes in risk factors over time.

## Results

### Heterosexual men (MSW)

**Figure 1:**
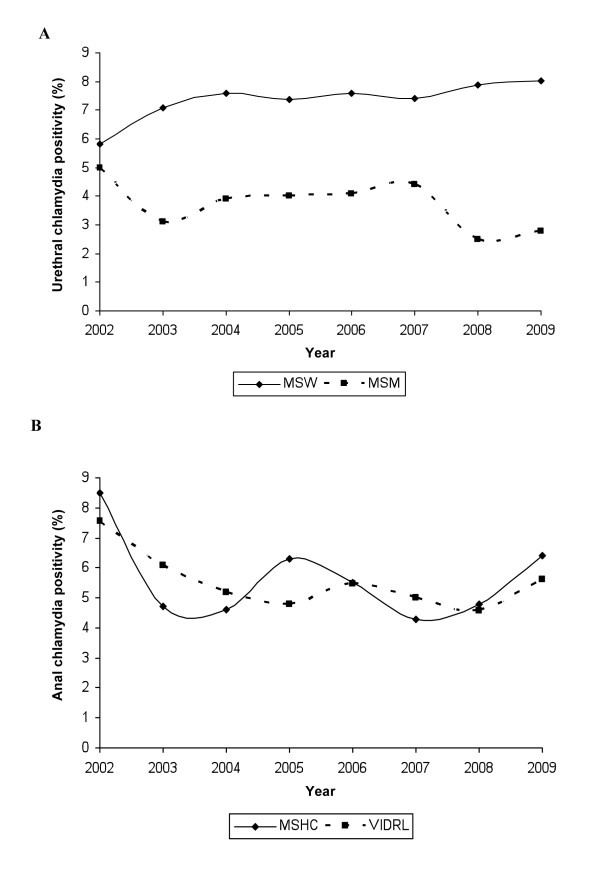
**Urethral and anal chlamydia positivity**. Changes in urethral chlamydia positivity over time in MSW and MSM (**A**) and in anal chlamydia positivity over time in MSM (**B**) at the Melbourne Sexual Health Centre (MSHC) and the Victorian Infectious Disease Reference Laboratory (VIDRL). MSW = men who have sex with women, MSM = men who have sex with men. NB: For 2002 and 2009, MSHC data are only available for a 6 month time period July-Dec 2002 and Jan-June 2009.

There were 17769 chlamydia tests conducted among MSW presenting for the first time during the time period. The median age of men tested for urethral chlamydia was 29 years (range 13-85 years). Overall chlamydia positivity for all MSW tested for chlamydia during the time period was 7.37% (95% CI: 6.99-7.77) and increased from 5.80% in 2002 to 8.02% in 2009 (Figure 1A).

Univariate analysis demonstrated a 3% greater odds of chlamydia positivity from one year compared with the previous year (OR = 1.03; 95% CI: 1.01-1.06; p = 0.03) (Table 1). Multivariate analysis adjusting for age, number of sexual partners in the last 12 months, condom use in the last 3 months, presence of symptoms and contact with infection, found a 4% greater odds of chlamydia positivity from one year compared with the previous year (OR = 1.04; 95% CI: 1.01-1.07; p = 0.02).

**Table 1:** Chlamydia positivity and associations in men who have sex with women (MSW) attending MSHC 2002-2009

Variable	Number tested	Number positive (%)	Crude OR (95% CI)	**Adjusted OR**^**b **^**(95% CI)**
**All MSW**	17769	1310 (7.37)		
**Year of test**^a ^				
			1.03 (1.01-1.06)	1.04 (1.01-1.07)
**Age group**				
< 25	4310	433 (10.05)	1.00	1.00
25-34	7908	632 (7.99)	0.78 (0.68-0.88)	0.79 (0.68-0.91)
> 35	5549	245 (4.42)	0.41 (0.35-0.49)	0.39 (0.32-0.46)
**No. female sexual partners 12 mo**				
0-1	4263	172 (4.03)	1.00	1.00
> 1	12698	1089 (8.58)	2.23 (1.89-2.63)	2.11 (1.75-2.5)
**Condom use 12 mo**				
always	2583	88 (3.41)	1.00	
sometimes, usually, never	13883	1167 (8.41)	2.60 (2.09-3.24)	
**No. female sexual partners 3 mo**				
0-1	9499	565 (5.95)	1.00	
> 1	7470	696 (9.32)	1.62 (1.45-1.82)	
**Condom use 3 mo**				
always	3024	110 (3.64)	1.00	1.00
sometimes, usually, never	12277	1117 (9.10)	2.65 (2.17-3.24)	2.78 (2.23-3.46)
**Contact with infection or symptomatic presentation**				
No	6546	258 (3.94)	1.00	1.00
Yes	8650	937 (10.83)	2.96 (2.57-3.41)	3.34 (2.89-3.88)

^a ^The odds ratio (OR) shows the association with chlamydia with each additional calendar year.

^b ^Adjusted for age, number of sexual partners last 12 months, condom use last 3 months and contact history or symptomatic presentation.

OR = odds ratio, MSHC Melbourne Sexual Health Centre, mo = months

The proportion of men reporting two or more sex partners in the last 3 months increased from 41.8% in 2002 to 52.9% in 2009 (p < 0.01) and, in the last 12 months from 73.0% in 2002 to 80.0% in 2009 (p < 0.01), but there was negligible change in condom use (p = 0.42 for condom use over 3 months and p = 0.40 for condom use over 12 months). The proportion of men reporting symptomatic presentation or contact with infection decreased from 59.4% in 2002 to 55.9% in 2009 (p < 0.01).

### Men who have sex with men (MSM)

There were 7977 tests for urethral and 6237 tests for anal chlamydia (8328 clients) and 5833 anal and 6980 pharyngeal tests (7133 clients) for gonorrhea conducted among MSM. The median age of men tested was 31 years (range 15-84 years), a similar profile to MSW attending the clinic during the same time.

#### Chlamydia in MSM

Overall positivity for urethral chlamydia in all MSM tested during the time period was 3.70% (95% CI: 3.30-4.14) and for anal chlamydia was 5.36% (95% CI: 4.82-5.96) and neither changed significantly between 2002 and 2009 (Figures 1A&B).

Univariate analysis found weak evidence to support a decrease in the odds of urethral chlamydia positivity (OR = 0.95; 95% CI: 0.90-1.01; p = 0.09) but no significant change in the odds of men testing positive for anal chlamydia over time (OR = 0.98; 95% CI: 0.93-1.03; p = 0.42) (Table 2). Multivariate analysis adjusting for age, number of sexual partners in the last 3 months, condom use in the last 12 months, presence of symptoms and contact with infection, showed no significant change in the odds of being positive for urethral chlamydia over time (OR = 0.95; 95% CI: 0.88-1.01; p = 0.12). Multivariate analysis adjusting for age, number of sexual partners in the last 3 months, condom use in the last 12 months, presence of symptoms and contact with infection, found no change in the odds of anal chlamydia positivity from one year compared to the previous year (OR = 0.99; 95% CI: 0.93-1.06; p = 0.76).

**Table 2:** Chlamydia positivity and associations in men who have sex with men (MSM) attending MSHC 2002-2009

	Urethral chlamydia				Anal chlamydia			
	
	Number tested	Number positive (%)	Crude OR (95% CI)	**Adjusted OR**^**b **^**(95% CI)**	Number tested	Number positive (%)	Crude OR (95% CI)	**Adjusted OR**^**b **^**(95% CI)**
**All MSM**	7977	292 (3.70)			6237	334 (5.36)		
**Year of test**^a^								
			0.95 (0.90-1.01)	0.95 (0.88-1.01)			0.98(0.93-1.03)	0.99 (0.93-1.06)
**Age group**								
< 25	1878	72 (3.83)	1.05 (0.78-1.42)	0.93 (0.62-1.38)	1565	82 (5.24)	1.18 (0.88-1.60)	1.30 (0.89-1.90)
25-34	3062	109 (3.56)	0.97 (0.74-1.27)	1.03 (0.74-1.44)	2453	153 (6.24)	1.42 (1.10-1.85)	1.53 (1.09-2.15)
> 35	3036	111 (3.66)	1.00	1.00	2218	99 (4.46)	1.00	1.00
**No. male sexual partners 12 mo**								
0-1	1439	53 (3.68)	1.00		824	31 (3.76)	1.00	1.00
> 1	5672	211 (3.72)	1.01 (0.74-1.37)		4635	256 (5.52)	1.50 (1.02-2.19)	1.78 (1.06-3.00)
**Condom use 12 mo**								
always	2615	78 (2.98)	1.00	1.00	2187	85 (3.89)	1.00	
sometimes, usually, never	3363	153 (3.98)	1.55 (1.17-2.05)	1.55 (1.14-2.11)	2963	204 (6.88)	1.83 (1.41-2.37)	
**No. male sexual partners 3 mo**								
0-1	2849	96 (3.37)	1.00	1.00	1970	86 (4.37)	1.00	
> 1	4449	177 (3.98)	1.19 (0.92-1.53)	1.40 (1.00-1.95)	3661	213 (5.82)	1.35 (1.05-1.75)	
**Condom use 3 mo**								
always	2564	85 (3.32)	1.00		2205	93 (4.22)	1.00	1.00
sometimes, usually, never	2920	135 (4.62)	1.41 (1.07-1.86)		2590	182 (7.03)	1.72 (1.33-2.22)	1.88 (1.40-2.52)
**Contact with infection or symptomatic presentation**								
No	3169	61 (1.92)	1.00	1.00	2555	118 (4.62)	1.00	1.00
Yes	3008	174 (5.78)	3.13 (2.33-4.21)	2.79 (2.02-3.86)	2112	137 (6.49)	1.43 (1.11-1.85)	1.43 (1.08-1.89)

^a ^The odds ratio (OR) shows the association with chlamydia with each additional calendar year.

^b ^Adjusted for age, number of sexual partners last 12 months, condom use last 3 months and contact history or symptomatic presentation.

OR = odds ratio, MSHC Melbourne Sexual Health Centre, mo = months

Among MSM testing for chlamydia, the proportion of men reporting symptoms or contact with infection at the time of testing decreased slightly from 39.1% in 2002 to 35.0% in 2009 (p < 0.01), but there was negligible change in the proportion reporting two or more sexual partners (p = 0.40 for partners last 3 months and p = 0.57 for partners in last 12 months) or condom use in either the last 3 or 12 months (p = 0.23 for condom use over 3 months and p = 0.95 for condom use over 12 months).

Over the same period there were 14083 anal swabs submitted to the VIDRL laboratory of which 752 (5.3%; 95% CI: 4.98-5.73) were positive, a similar overall positivity rate to that diagnosed at MSHC (p = 0.96). Similarly, swabs tested for chlamydia at VIDRL showed a significant decrease in the odds of positivity from one year to the previous year (OR = 0.97; 95% CI: 0.93-0.99; p = 0.05) (Additional file 1 Figure 1B). The number of anal swabs submitted to the laboratory rose steadily from 858 in 2002 to 2875 in 2009 (p < 0.01).

#### Gonorrhea in MSM

**Figure 2:**
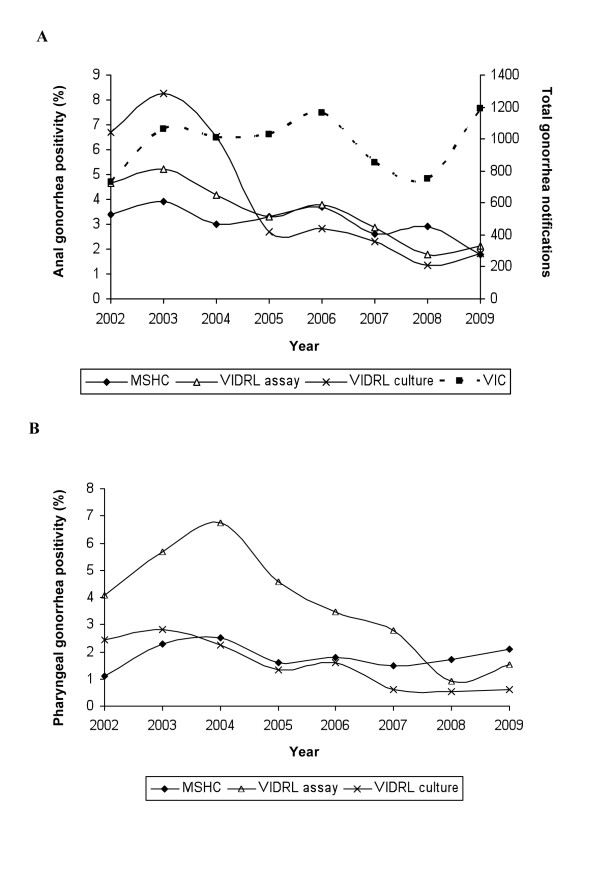
**Anal and pharyngeal gonorrhea positivity**. Changes in anal (**A**) and pharyngeal (**B**) gonorrhea positivity over time at both Melbourne Sexual Health Centre (MSHC) and the Victorian Infectious Disease Reference Laboratory (VIDRL; two types of sampling methods; nucleic acid amplification assay and culture). The total number of notified cases of gonorrhea in Victorian (VIC) men between 2002 and 2009 are also indicated (**A**). NB: For 2002 and 2009, MSHC data are only available for a 6 month time period July-Dec 2002 and Jan-June 2009.

Overall positivity for anal gonorrhea for all MSM included in the analysis was 3.05% (95% CI: 2.63-3.53) and for pharyngeal samples was 1.83% (95% CI: 1.53-2.18) (Figures 2A&B). Anal gonorrhea positivity decreased over time from 3.44% in 2002 to 1.81% in 2009 but there was no change in pharyngeal gonorrhea positivity over time.

Univariate analysis demonstrated a borderline significant decline in the odds of men testing positive for anal gonorrhea from one year to the previous year (OR = 0.93; 95% CI: 0.87-1.00; p = 0.05) but not for pharyngeal gonorrhea (OR = 0.99; 95% CI: 0.91-1.07; p = 0.77) (Table 3). Multivariate analysis adjusting for age, number of sexual partners in the last 3 months, condom use, presence of symptoms and contact with infection, found no change in the odds of being positive for either anal or pharyngeal gonorrhea over time (OR = 0.99; 95% CI: 0.89-1.10; p = 0.27 and OR = 0.99; 95% CI: 0.91-1.11; p = 0.79, respectively).

**Table 3:** Gonorrhea positivity and associations in men who have sex with men (MSM) attending MSHC 2002-2009

	Anal Gonorrhea				Pharyngeal Gonorrhea			
	
	Number tested	Number positive (%)	Crude OR (95% CI)	**Adjusted OR**^**b **^**(95% CI)**	Number tested	Number positive (%)	Crude OR (95% CI)	**Adjusted OR**^**b **^**(95% CI)**
**All MSM**	5833	178 (3.05)			6980	128 (1.83)		
**Year of test**^a ^								
			0.93 (0.87-1.00)	0.95 (0.88-1.04)			0.99 (0.91-1.07)	1.01 (0.93-1.11)
**Age group**								
< 25	1496	62 (4.14)	2.28 (1.44-3.60)	2.46 (1.54-3.94)	1735	47 (2.71)	1.00	2.68 (1.63-4.41)
25-34	2314	68 (2.94)	1.56 (0.99-2.45)	1.54 (0.96-2.46)	2677	50 (1.87)	0.68 (0.46-1.02)	1.70 (1.05-2.78)
> 35	2022	48 (2.37)	1.00	1.00	2567	31 (1.22)	0.44 (0.28-0.69)	1.00
**No. male sexual partners 12 mo**								
0-1	784	11 (1.40)	1.00	1.00	965	10 (1.04)	1.00	
> 1	4384	144 (3.28)	2.39 (1.29-4.43)	1.83 (0.91-3.64)	5314	107 (2.01)	1.96 (1.02-3.77)	
**Condom use 12 mo**								
always	2093	48 (2.29)	1.00					
sometimes, usually, never	2761	112 (4.06)	1.80 (1.28-2.54)					
**No. male sexual partners 3 mo**								
0-1	1861	36 (1.93)	1.00		2261	29 (1.28)	1.00	1.00
> 1	3465	126 (3.64)	1.91 (1.32-2.78)		4187	94 (2.25)	1.77 (1.16-2.69)	1.63 (1.05-2.54)
**Condom use 3 mo**								
always	2107	54 (2.56)	1.00	1.00				
sometimes, usually, never	2414	101 (4.18)	1.66 (1.19-2.32)	1.64 (1.13-2.39)				
**Contact with infection or symptomatic presentation**								
No	2425	41 (1.69)	1.00	1.00	2876	29 (1.01)	1.00	1.00
Yes	1960	113 (5.77)	3.56 (2.48-5.11)	3.63 (2.45-5.38)	2468	85 (3.44)	3.50 (2.29-5.36)	3.69 (2.40-5.68)

^a ^The odds ratio (OR) shows the association with chlamydia with each additional calendar year.

^b ^Adjusted for age, number of sexual partners last 12 months, condom use last 3 months and contact history or symptomatic presentation.

OR = odds ratio, MSHC Melbourne Sexual Health Centre, mo = months

Among MSM testing for gonorrhea, the proportion of men reporting two or more sex partners decreased from 66.60% to 63.5% for 3 months (p < 0.01) and from 87.6% to 83.4% for 12 months (p < 0.01), but there was negligible change in condom use (p = 0.52). The proportion of men reporting contact with infection or symptoms decreased slightly from 49.0% in 2002 to 46.4% in 2008, before rising again in 2009 to 49.0% (p = 0.04).

Over the same period there were 14083 anal swabs from MSM submitted to VIDRL for gonorrhea testing by PCR and 10526 swabs submitted for culture of which 432 (3.07%; 95% CI: 2.79%-3.37%) and 361 (3.43%; 95% CI: 3.09%-3.80%) were positive, respectively. Swabs tested by PCR for gonorrhea showed a significant decrease in the odds of positivity (OR = 0.86; 95% CI: 0.83-0.90; p < 0.01) (Additional file 1, Figure 2A). Similarly, swabs tested by culture for gonorrhea showed a significant decrease in the odds of positivity (OR = 0.73; 95% CI: 0.73-0.80; p < 0.01). The number of anal swabs submitted for both PCR and culture rose between two to three fold between 2002 and 2009 (Additional file 1) (p < 0.01).

There were 2270 pharyngeal swabs submitted to VIDRL for gonorrhea testing by PCR and 18,355 swabs submitted for culture of which 89 (3.92%; 95% CI: 3.18%-4.82%) and 239 (1.30%; 95% CI: 1.14%-1.48%) were positive respectively. Swabs tested by PCR for gonorrhea showed a significant decrease in the odds of being positive for pharyngeal gonorrhea (OR = 0.86; 95% CI 0.78-0.95; p < 0.01) (Additional file 1, Figure 2B). Similarly swabs tested by culture for gonorrhea showed a significant decrease in the odds of positivity (OR = 0.77; 95% CI 0.73-0.82; p < 0.01) (Additional file 1 Figure 2B).

There were 283 cases of urethral gonorrhea diagnosed at MSHC during the study period. The odds of urethral gonorrhea positivity decreased over the time period (OR = 0.83; 95% CI: 0.78-0.88; p < 0.01) (Table 4).

**Table 4:** Urethral gonorrhea positivity in men who have sex with men (MSM) attending MSHC 2002-2009

Year	Number tested^a ^	Number positive (%)
2002	632	19 (3.00)
2003	1104	84 (7.61)
2004	909	43 (4.73)
2005	931	32 (3.44)
2006	1007	46 (4.57)
2007	1055	21 (1.99)
2008	1286	28 (2.18)
2009	687	10 (1.46)
OR (95% CI)*		0.83 (0.78, 0.88)
*p-value*		*p < 0.01*

^a ^Number tested includes all MSM who were tested for gonorrhea at any site

NB: For 2002 and 2009, MSHC data are only available for a 6 month time period July-Dec 2002 and Jan-June 2009. OR = odds ratio, CI = confidence interval.

*Odds ratio for the association of gonorrhea positivity with year

Notifications per annum for gonorrhea in men between 2002 and 2009 in Victoria were stable over time (p = 0.63) (Figure 2A).

## Discussion

We found that chlamydia positivity increased significantly among MSW attending MSHC over the time period after adjustment for known risk factors. In contrast, we found weak evidence to support a small decrease in urethral chlamydia positivity and no change in anal chlamydia positivity among MSM. We also found that there was some evidence to suggest that anal gonorrhea positivity among MSM declined during the same period. This change may be explained by changes in risk behaviors. The percentage of men infected with urethral gonorrhea also declined significantly at MSHC over the same period. Data from the principle infectious diseases laboratory for Melbourne, VIDRL, supported our clinic findings showing significant declines in anal and pharyngeal gonorrhea positivity over time and a less marked, but also significant, decline in anal chlamydia. It is important to note that no adjustment for behavioral risk was possible with the VIDRL data. Taking the MHSC, VIDRL and Victorian gonorrhea notification data into consideration, it appears that gonorrhea and chlamydia rates in MSM are either stable or declining while chlamydia positivity among MSW appears to be rising.

A rise in chlamydia positivity in MSW is consistent with our previously reported increase in chlamydia positivity in women attending MSHC. Community based studies report similar prevalence estimates of 3 to 5% among young men and women alike in Australia [15-18]. Other studies have also reported increasing chlamydia positivity [5,6,19-24]. Surveillance data of the proportion of chlamydia tests positive from Sweden, Denmark, Ireland and the US [23-29] have also shown increasing positivity. Sexual behavior data show that sexual risk behavior is also changing with decreasing age at first sex [30] and increasing number of annual sex partners [31]. In this context, it appears that a true increase in chlamydia infections among both women and MSW appears likely, and that even the relatively high rates of screening in the Scandinavian countries of about 25% per annum in women are insufficient to arrest this rise [21].

While our analysis suggests that gonorrhea and chlamydia infection rates in MSM are stable or possibly declining, the rates of syphilis and HIV in MSM have been rising. In 2002 there were 22 cases of infectious syphilis in Victoria compared with 391 in 2009 and almost all cases occurred in MSM [32]. In 2002 there were 165 newly diagnosed cases of HIV in men compared with 238 in 2008 with over 80% of male cases in MSM [2]. These increasing HIV and syphilis rates do provide some evidence to suggest that sexual behavior in the population may have changed and this is supported by evidence from community based surveys of MSM showing increases in sexual risk such as declining condom use [11,33]. HIV and syphilis are different infections from chlamydia and gonorrhea and likely to have different transmission dynamics requiring different testing frequencies. In this context, the declining gonorrhea and chlamydia positivity observed in this study in combination with increasing sexual risk behavior over time, does provide further evidence that gonorrhea and chlamydia testing frequency among MSM may have reached a level where we might start seeing control or even decreasing community prevalence of these infections.

In MSM, very few studies have assessed the chlamydia and gonorrhea positivity over time. Marcus et al. reported positivity rates at several clinics in San Francisco from 2005 to 2008, and found no change in either anal chlamydia or gonorrhea positivity [34]. However, this study did not adjust for sexual risk factors or symptomatic presentations. The authors attributed the stable rates of positivity to a significant increase in STI testing rates [34], supporting our hypothesis that increased testing reduces positivity rates in MSM. In contrast to our findings, an Australian MSM cohort study has reported high incidences of urethral and anal chlamydia and gonorrhea. But this study followed a single cohort of MSM over time and did not include any new people being tested [35].

There are a number of limitations in the analysis of MSHC data. Firstly it is a retrospective analysis of records from a sexual health clinic and may not be representative of changes in prevalence in the population. However, MSHC does a significant proportion of testing for MSM and in 2008 for example, diagnosed about 21% of Victorian gonorrhea cases [36]. Secondly, we have analyzed the proportion of individuals who tested positive each year rather than following a single cohort over time. However, cohort studies are expensive and difficult to conduct. In the absence of cohort studies, analyses of medical records can be accepted for investigating STI epidemiology over time. Finally, because data was only available from July 2002 and the analysis ended on the 30^th ^of June 2009, it is possible that a seasonal variation may have influenced our analysis. We therefore undertook a univariate analysis for equal 12 month periods from 1^st ^of July 2002 onwards. The results of this analysis were not different from the analysis by year of diagnosis (data not shown).

Finally, we were unable to evaluate all possible factors that may have affected the positivity over time. We did not measure the clients' sexual network characteristics such as concurrency during this time period which may be important in influencing an individual's risk of infection [37]. We did however, measure other factors and any changes were adjusted for in the analysis.

There were also a number of limitations for the data from VIDRL. Firstly, these data do include some men testing multiple times. However, the overall chlamydia and gonorrhea positivity estimates were similar to those at MSHC. Secondly, we have no data on the risk profile of patients testing. However the decline in the proportion positive, particularly for gonorrhea, was so striking that it is unlikely that these factors or unmeasured confounding could disguise a true increase in the community prevalence of either infection.

Our data from MSHC have a number of strengths. The analysis involved many men with either male or female sexual partners tested over a number of years. For chlamydia, the same laboratory and same diagnostic testing methods were used throughout the study period. For gonorrhea, both PCR and culture were used; however, only culture was used at MSHC during the study period and while both PCR and culture were used at VIDRL, the analysis was stratified by test type, to account for any difference in trends that may have been observed due to different test sensitivities. Furthermore, the same testing criteria were in place during the study period. The study is also strengthened by inclusion of the VIDRL data. While these data do lack epidemiological risk profiles, the specimens came from the same two large MSM clinics in Melbourne over the entire study period and give further support to the trends identified in the MSHC data.

The clear difference in trends between MSW, where chlamydia is rising, and MSM, where chlamydia and gonorrhea are stable or possibly decreasing, suggests that there may be different factors operating in these two groups. Data from both MSW and MSM in Australia and internationally suggest their sexual risk profile is changing with an increase in higher risk practices. Further, in Australia there is evidence that community antibiotic use is decreasing. Three Australian studies have reported lower chlamydia rates among individuals with recent antibiotic use [38-40]. Both changing sexual behavior and antibiotic use should put upward pressure on STI rates for MSW and MSM, yet this is not observed in MSM. One possible reason for the difference in chlamydia trends between MSW and MSM is the differential rates of STI testing. Only a small minority (less than 12% of 15-24-year-old women and less than 5% of 15-24-year-old) [4] of heterosexuals in Australia have had a chlamydia test in the last year while about half of MSM report being tested [21]. This raises the rather tantalizing possibility that we may have reached the point where the reproductive number for chlamydia and gonorrhea infection among MSM, may be at the point of falling as a result of increased testing.

## Conclusions

Overall, it appears that chlamydia positivity is increasing among MSW and chlamydia and gonorrhea positivity among MSM is remaining stable, suggesting that the epidemic of these two STIs may be equilibrating among MSM but not in MSW. We therefore hypothesize that increased testing could be having an impact on the transmission of chlamydia and gonorrhea in the population of MSM and that overall low rates of testing are contributing to the ongoing rise of heterosexually transmitted chlamydial infections in Australia.

## Competing interests

The authors declare that they have no competing interests.

## Authors' contributions

LAV participated in study design, carried out data collection and drafted the manuscript. CKF and JSH conceived the study and participated in the design of the study and drafting of the manuscript. GF also participated in study design and carried out data collection. Statistical analysis was performed by LAV, GF and JSH. DL carried out the data collection at VIDRL and assisted with drafting the manuscript. JW and CB participated in design of the study and helped to draft the manuscript. All authors read and approved the final manuscript.

## Pre-publication history

The pre-publication history for this paper can be accessed here:

http://www.biomedcentral.com/1471-2334/11/158/prepub

## Supplementary Material

Additional file 1 **Anal chlamydia and anal and pharyngeal gonorrhea positivity for men who have sex with men at MSHC and VIDRL by year**. Click here for file
